# Circulating tumor cells in the cerebrospinal fluid: “tapping” into diagnostic and predictive potential

**DOI:** 10.18632/oncotarget.349

**Published:** 2011-11-06

**Authors:** Bishoy Faltas

**Affiliations:** Department of Medicine, Rochester General Hospital, 1425 Portland Avenue, Rochester, NY 14621

Metastatic spread of breast cancer into the central nervous system (CNS) carries an especially ominous prognosis. Successful treatment depends on early and accurate diagnosis [1]. Therefore, it is of paramount clinical importance to develop sensitive and specific methods to detect circulating tumor cells (CTCs) in the cerebrospinal fluid (CSF). Routine cytological examination of the cerebrospinal fluid for tumor cells has many limitations including low sensitivity necessitating repeated lumbar punctures to increase the diagnostic yield [2]. In the October issue of Oncotarget, Patel *et al* developed a method to adapt the FDA approved CellSearch platform to isolate CTCs from the CSF after spiking them into blood [3]. This novel approach adds a “molecular dimension” to the detection of malignant epithelial cells in the CSF. It hones in on single cells and begins to characterize their surface markers thus opening the door to a better understanding of their role in the metastatic cascade. The CellSearch system defines CTCs as EpCAM+, Cytokeratin+, CD45- nucleated cells. These cells, when isolated from the CSF, may represent a special subpopulation of CTCs capable of surviving the perilous journey in the blood stream and subsequent invasion of the CNS. The next logical step in understanding the biology of this unique cell population will be to search for markers that would predict special affinity for homing into and invading the CNS, surviving and proliferating (i.e. stem cell markers) or for drug resistance [4]. Studying ER/PR and HER-2 concordance between primary or metastatic tumor sites and CTCs in the CSF could also have profound implications. Further studies are needed to create surface marker and genetic “profiles” of this important subset of CTCs. This study also demonstrated correlation between CTC counts in the CSF and the administration of chemotherapeutic agents, a pattern that mirrors that observed with CTCs in the peripheral blood of patients with metastatic breast cancer. Previous studies have confirmed the validity of circulating tumor cell counts in predicting progression free survival (PFS) and overall survival (OS) in patients with metastatic breast cancer. For instance, in one study of metastatic breast cancer, patients with more than 5 CTCs had lower PFS (2.7 months vs. 7.0 months, P<0.001) and shorter OS (10.1 months vs. >18 months, P<0.001). These differences persisted at the first follow-up visit after the initiation of therapy [5]. It would be interesting to see if larger studies confirm similar prognostic and predictive correlation between CTC counts in the CSF and survival or response to therapy. Our understanding of the role of CTCs in the biology of metastases continues to evolve. This valuable study sheds the light on a subset of CTCs capable of invading the blood brain barrier to reach the CNS. Further characterization and profiling of CTCs in the CSF is likely to yield valuable insights into the biology and the behavior of this important subset of cells and to improve our understanding of how CNS metastases occur. Correlation between CTCs counts, their biological characteristics and clinical outcomes will continue to make this area both promising and clinically relevant.
